# *Zygosaccharomyces rouxii* Combats Salt Stress by Maintaining Cell Membrane Structure and Functionality

**DOI:** 10.4014/jmb.1904.04006

**Published:** 2019-10-13

**Authors:** Dingkang Wang, Min Zhang, Jun Huang, Rongqing Zhou, Yao Jin, Chongde Wu

**Affiliations:** 1 College of Light Industry, Textile and Food Engineering, Sichuan University, Chengdu 60065, P.R. China; 2 Key Laboratory of Leather Chemistry and Engineering, Ministry of Education, Sichuan University, Chengdu 610065, P.R. China

**Keywords:** Salt stress, *Zygosaccharomyces rouxii*, cell membrane, structure, functionality

## Abstract

*Zygosaccharomyces rouxii* is an important yeast that is required in the food fermentation process due to its high salt tolerance. In this study, the responses and resistance strategies of *Z. rouxii* against salt stress were investigated by performing physiological analysis at membrane level. The results showed that under salt stress, cell integrity was destroyed, and the cell wall was ruptured, which was accompanied by intracellular substance spillover. With an increase of salt concentrations, intracellular Na^+^ content increased slightly, whereas intracellular K^+^ content decreased significantly, which caused the increase of the intracellular Na^+^/K^+^ ratio. In addition, in response to salt stress, the activity of Na^+^/K^+^-ATPase increased from 0.54 to 2.14 μmol/mg protein, and the ergosterol content increased to 2.42-fold to maintain membrane stability. Analysis of cell membrane fluidity and fatty acid composition showed that cell membrane fluidity decreased and unsaturated fatty acid proportions increased, leading to a 101.21% rise in the unsaturated/saturated fatty acid ratio. The results presented in this study offer guidance in understanding the salt tolerance mechanism of *Z. rouxii*, and in developing new strategies to increase the industrial utilization of this species under salt stress.

## Introduction

During the production of fermented foods, microorganisms encounter various stress conditions, and salt stress is one of the main challenges for their survival. In a hyperosmotic environment, microorganisms develop several strategies to overcome salt stress, including regulation of cellular metabolism, accumulation of compatible compounds, and activation of transporters [1].

The cell membrane is considered to be the first barrier that separates a cell from its environment and is the primary target for damage induced by environmental stress [2]. To resist salt stress, several alterations in the structure and functionality of the cell membrane have been observed, mainly including cell membrane integrity, Na^+^/K^+^-ATPase activity, fluidity, and unsaturated fatty acid (UFA) proportion, etc. Cell integrity plays a significant role in maintaining cell viability and metabolic functions under environmental stress. In general, maintaining the integrity of cell membrane could prevent cell death at the onset of salt stress [3]. Na^+^/K^+^-ATPase, a protein embedded in the lipid bilayer of the cytoplasmic membrane, catalyzes ATP hydrolysis to provide energy, drives Na^+^ and K^+^ between both sides of cell membranes, and maintains cell osmotic pressure to provide energy for nutritive absorption [4]. Alterations in cell membrane fluidity may be related to salt tolerance. Previous research has demonstrated that high cell membrane fluidity and a homogeneous distribution of fluidity values appeared to be positively related to freeze-thaw resistance [5]. Regarding ethanol stress, Ishmayana *et al*. [6] have suggested that lower membrane fluidity led to higher ethanol tolerance. In addition, salt tolerance is reported to be closely related to the membrane lipid compositions; Turk *et al*. [7] revealed that salt stress causes an increase in fatty acid unsaturation in the halophilic *Hortaea werneckii* and halotolerant *Aureobasidum pulluans*. Furthermore, Sara *et al*. [8] found that supplemented oleic acid and ergosterol would mitigate oxidative stress in wine strains of *Saccharomyces cerevisiae*.

*Zygosaccharomyces rouxii* exhibits high salt tolerance and is considered an important yeast in the food fermentation process [9]. The cell membrane of *Z. rouxii* plays an important role in cellular growth, metabolism, and energy transduction. However, the behavior of *Z. rouxii* in maintaining cell membrane structure and functionality to combat salt stress has not been sufficiently explored. Therefore, in this study, we performed a comprehensive analysis of the cell membrane responses of *Z. rouxii* to salt stress.

## Materials and Methods

### Strains and Salt Stress Experiment 

*Z. rouxii* CGMCC 3791 was isolated from soy sauce and identified by 26s rDNA sequencing analysis. Inocula were transferred from −80°C frozen stock to YPD medium (1% yeast extract, 2% peptone and 2% glucose, pH 6.0), and then incubated statically at 30°C for 24 h as pre-cultures. To investigate the growth performance of *Z. rouxii*, the pre-culture was incubated with an inoculum size of 5% (vol/vol) to 100 ml of YPD medium with different NaCl concentrations (0%, 6%, 12%, and 18%) and cultured at 30°C. Cells grown to the mid-exponential growth phase in different salt (NaCl) concentrations were harvested, centrifuged at 10,000 g for 5 min, and washed twice serially with saline water for subsequent analyses.

### Transmission Electron Microscopy (TEM) Analysis

Samples for TEM analysis were prepared by slightly modifying the method described by Wu *et al*. [10]. The differences included that samples were fixed in 2.5% (v/v) glutaraldehyde for 3 h and the cell pellets were mixed with 1% (w/v) water agar. Epon 812 (SPI Supplies, USA), comprising cell agar slices, was sliced into 80-nm sections by using a Leica EM UC7 ultramicrotome (Leica Microsystems, Austria). These thinly sliced sections were stained with uranyl acetate and then lead citrate prior to examination under a JEM 2100 Plus electron microscope (JEOL, Japan).

### Determination of Intracellular Na^+^ and K^+^ Concentrations

Intracellular Na^+^ and K^+^ concentrations were determined according to the method described by González-Hernández *et al*.[11]. The cells were disrupted by incubation with 200 µM cetyltrimethylammonium bromide for 15 min at room temperature and the suspension was then centrifuged to obtain the samples. The samples were analyzed by flame atomic absorption spectroscopy (SpectrAA-220FS, VARIAN, USA).

### Analysis of Na^+^/K^+^-ATPase Activity

Na^+^/K^+^-ATPase activity was evaluated by measuring the release of inorganic phosphate (Pi) from ATP according to the kit protocol (Nanjing Jiancheng Bioengineering Institute, China). Briefly, the bacterial suspensions were sonicated on ice (20 kHz, 25 min) and centrifuged at 10,000 g for 5 min to obtain the supernatant. The Na^+^/K^+^-ATPase activity of the supernatant was determined by measuring the amount of Pi using the malachite green dye method and expressed as units per milligram of protein [12]. Protein concentration was determined according to the Bradford method by using bovine serum albumin as the standard protein.

### Determination of Ergosterol Concentration

Ergosterol concentration was measured according to the method described by Alizadeh *et al*. [13]. Each cell mass was added to 3 ml of ethanol solution comprising 25% potassium hydroxide and was then incubated at 85°C for 1 h in a water bath. Subsequently, a mixture of 1 ml of sterile distilled water and 3 ml of n-heptane (Sigma-Aldrich, USA) were added, and the solution was vortexed for 3 min. The supernatant was obtained, and its absorbance was read at 282 and 230 nm using a spectrophotometer. A calibration curve was generated for standard ergosterol to calculate the ergosterol concentration, which was expressed as dry cell weight (DCW).

### Measurement of Cell Membrane Fluidity

Cell membrane fluidity was determined by fluorescence anisotropy, which was performed to study the rotational diffusion of fatty acyl chains in the membrane interior, according to the method described by Liao *et al*. [14]. The lipid-soluble fluorescent probe 1,6-diphenyl-1,3,5-hexatriene (DPH) was used to monitor the changes in membrane dynamics. Measurements were obtained using a spectrofluorometer (model 5J1-0042, Hitachi F-7100, Japan), with excitation at 360 nm and emission at 430 nm (5-nm slits, respectively). The calculation of fluorescence anisotropy referred to the method of Ansari *et al*. [15]. The degree of fluorescence polarization (*p*) and anisotropy (*r*) were calculated as follows:

$$p=\frac{\mathrm{IVV–IVH}(\mathrm{IHV⁄}\mathrm{IHH})}{\mathrm{IVV}+\mathrm{IVH}(\mathrm{IHV⁄}\mathrm{IHH})},r=\frac{2p}{3\mathrm{–p}}$$

where *I*v is the corrected fluorescence and the subscripts *V* and *H* represent the values obtained by the excitation and analyzer polarizers in the vertical and horizontal direction, respectively.

### Extraction and Analysis of Cell Membrane Fatty Acids

The extraction of membrane lipids and preparation of fatty acid methyl esters (FAMEs) were conducted according to the method described by Wu *et al*. [16]. The samples were analyzed using gas chromatography mass spectroscopy (GC-MS, Trace GC Ultra-DSQII, Thermo Electron Corporation, USA) according to a previously described method [17]. The relative amount of FAMEs was calculated from peak areas. The degree of unsaturation (unsaturated fatty acids/saturated fatty acids, U/S ratio) and the mean chain length were determined according to the previously described method [10]. All experiments were performed in triplicate.

### Statistical Analysis

One-way ANOVA with Duncan’s test was used to investigate the statistical differences. Differences between groups with *p* values less than 0.05 (*n* = 3), were considered to be statistically significant. One-way ANOVA was performed using the SPSS software (version 19.0, SPSS Inc., IBM, USA).

## Results

### TEM Analyses of *Z. rouxii* under Salt Stress

**Fig. 1:**
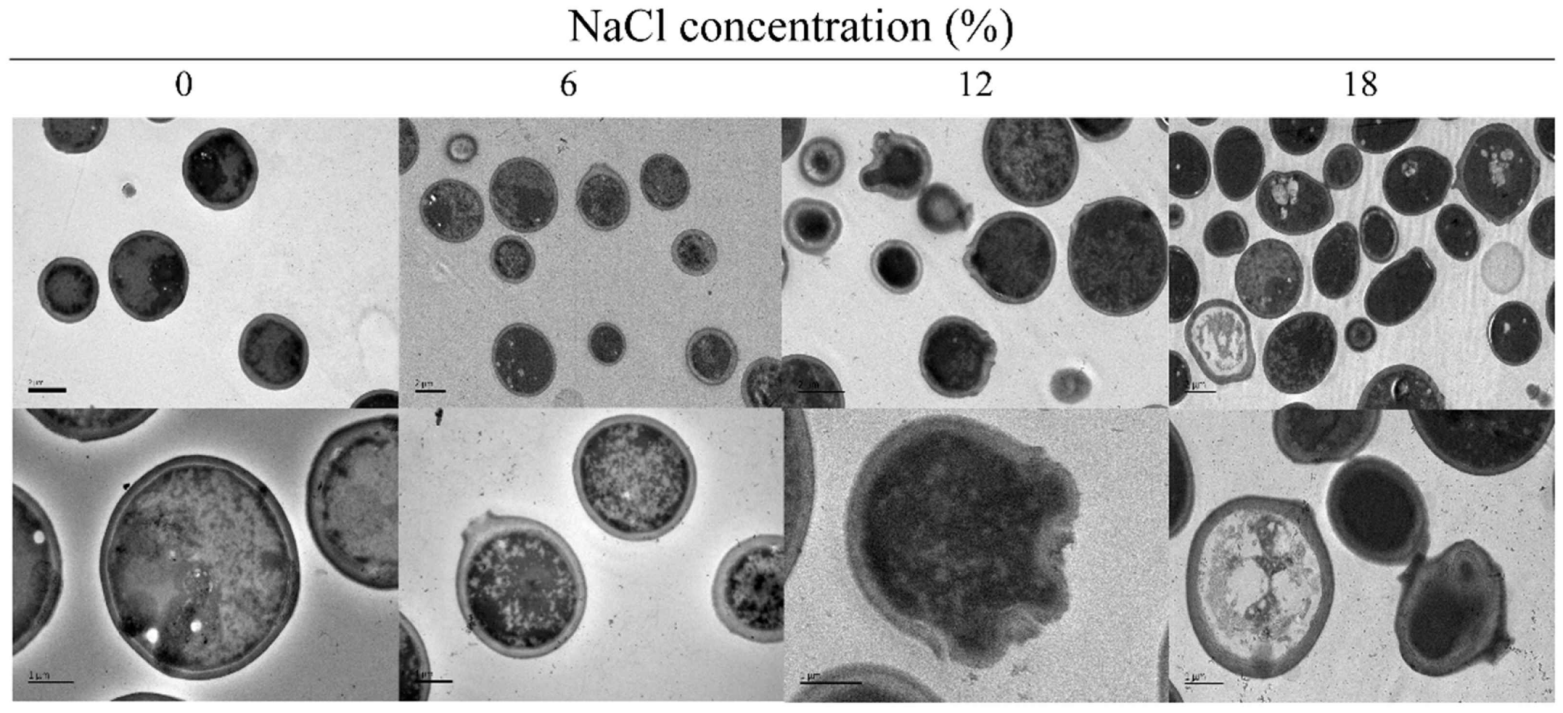
TEM analyses of the morphological changes in *Z. rouxii* growing to mid-exponential growth phase under salt stress (NaCl concentration: 0%, 6%, 12%, and 18%).

Salt-induced changes in the cell morphology of *Z. rouxii* were revealed by TEM analysis (Fig. 1). In the absence of NaCl, the cells were round, cell surface was smooth and cell walls were intact. Under salt stress, the cells transformed into an elliptical shape, and developed a rough surface, while the cell walls became relatively thinner and coarser. In addition, cell rupture was accompanied by intracellular substance spillover. These results showed that an increase in NaCl concentrations, affected *Z. rouxii* cells, including their cell shape, cell wall, cell membrane, and intracellular solute substances.

### Analysis of Intracellular Na^+^ and K^+^ Concentrations

**Fig. 2:**
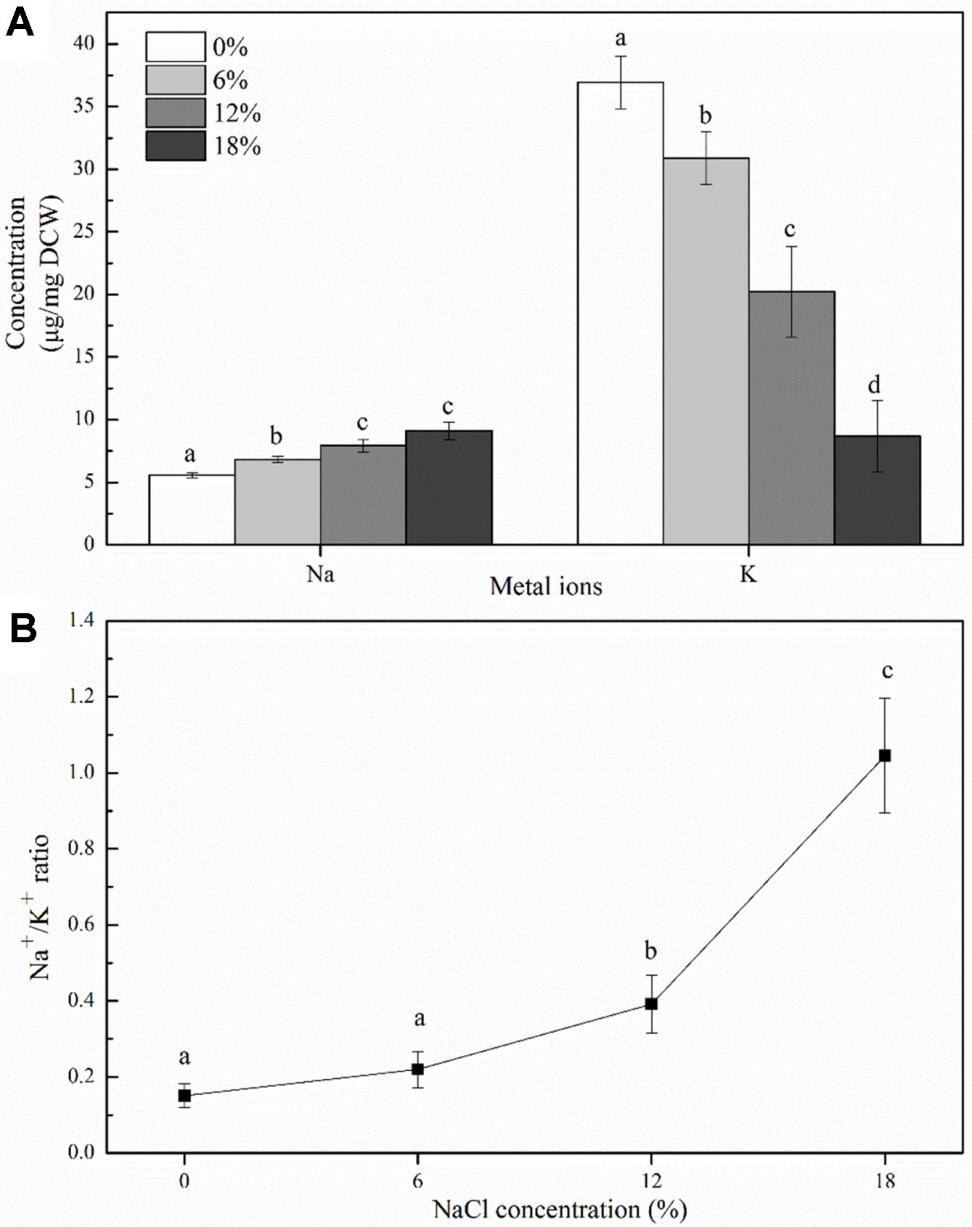
Changes of intracellular Na^+^, K^+^ concentrations (**A**) and Na^+^/K^+^ ratio (**B**) of *Z. rouxii* upon salt stress. Cells were cultured to mid-exponential growth phase under salt stress (NaCl concentration: 0%, 6%, 12%, and 18%) in YPD medium. *Error bars*: SD (*n* = 3). Statistically significant differences (*p* < 0.05) were determined by one-way ANOVA with Duncan’s test and were indicated with different letters.

Changes in the intracellular metal ion Na^+^ and K^+^ concentrations in *Z. rouxii* under salt stress were investigated (Fig. 2A). The results showed that with an increase in NaCl concentrations, intracellular Na^+^ concentrations increased gradually, from 0% NaCl concentration (at 5.57 ± 0.19 µg/mg DCW) to 18% NaCl concentration (at 9.11 ± 0.69 µg/mg DCW), whereas intracellular K^+^ concentration decreased from 36.94 ± 2.10 to 8.68 ± 2.86 µg/mg DCW. Meanwhile, the Na^+^/K^+^ ratio exhibited an increase tendency (Fig. 2B). These results suggested that salt stress caused an increase in intracellular content of Na^+^, and a decrease in content of K^+^ , thus eventually resulting in an increase in the Na^+^/K^+^ ratio.

### Changes in the Na^+^/K^+^-ATPase Activity of *Z. rouxii* under Salt Stress

**Fig. 3:**
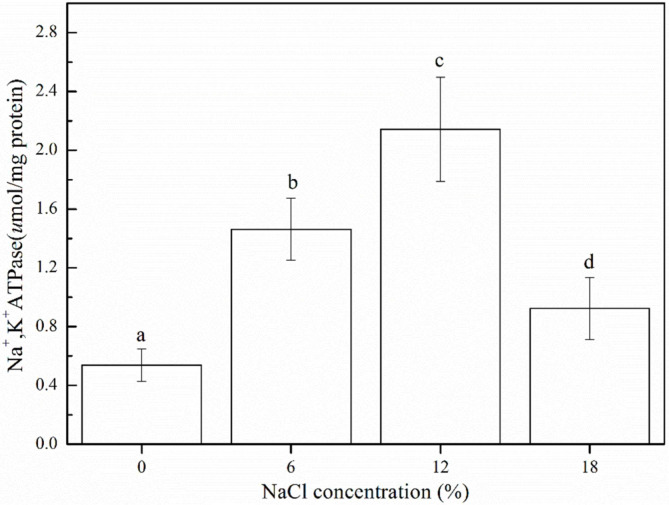
Changes in Na^+^-K^+^-ATPase activity expression of *Z. rouxii* under salt stress (NaCl concentration: 0%, 6%, 12%, and 18%). *Error bars*: SD (*n* = 3). Statistically significant differences (*p* < 0.05) were determined by one-way ANOVA with Duncan’s test and were indicated with different letters.

The results of Na^+^/K^+^-ATPase activity are presented in Fig. 3. In normal YPD medium (non-salt medium), the Na^+^/K^+^-ATPase activity of *Z. rouxii* was low, at 0.54 ± 0.11 µmol/mg protein. With increasing NaCl concentrations, Na^+^/K^+^-ATPase activity increased gradually, and the maximum activity (2.14 ± 0.35 µmol/mg protein) was obtained under 12% NaCl concentration. Na^+^/K^+^-ATPase activity decreased to 0.93 ± 0.21 µmol/mg protein when the NaCl concentration was further increased to 18%. These results showed that an increase in Na^+^/K^+^-ATPase activity is a common strategy used by *Z. rouxii* to counteract salt stress.

### Accumulation of Ergosterol in *Z. rouxii* under Salt Stress

**Fig. 4:**
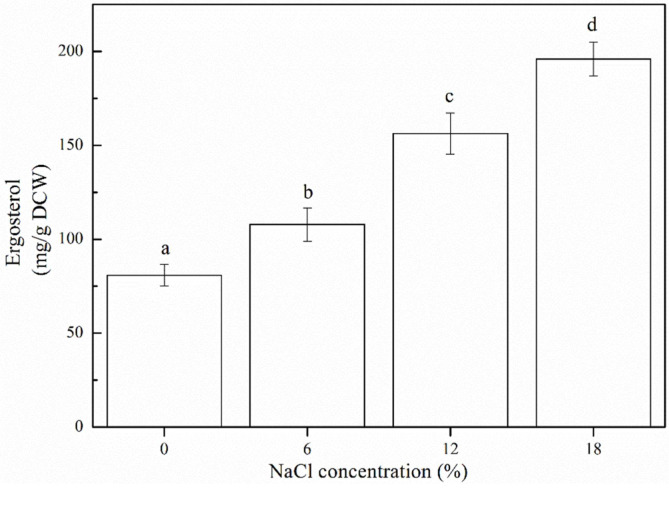
Accumulation of ergosterol by *Z. rouxii* in response to salt stress (NaCl concentration: 0%, 6%, 12%, and 18%). *Error bars*: SD (*n* = 3). Statistically significant differences (*p* < 0.05) were determined by one-way ANOVA with Duncan’s test and were indicated with different letters.

Ergosterol concentrations in *Z. rouxii* under different salt stress (NaCl) conditions are presented in Fig. 4. These concentrations increased with an increase in NaCl concentration, and the maximum accumulation of ergosterol (195.96 ± 8.96 mg/g DCW) was obtained at 18% NaCl. A 2.42-fold higher concentration was observed at 18% NaCl, than that observed in the absence of NaCl, thus suggesting that accumulation of ergosterol is a common response under salt stress.

### Changes in Cell Membrane Fluidity in *Z. rouxii* under Salt Stress

**Fig. 5:**
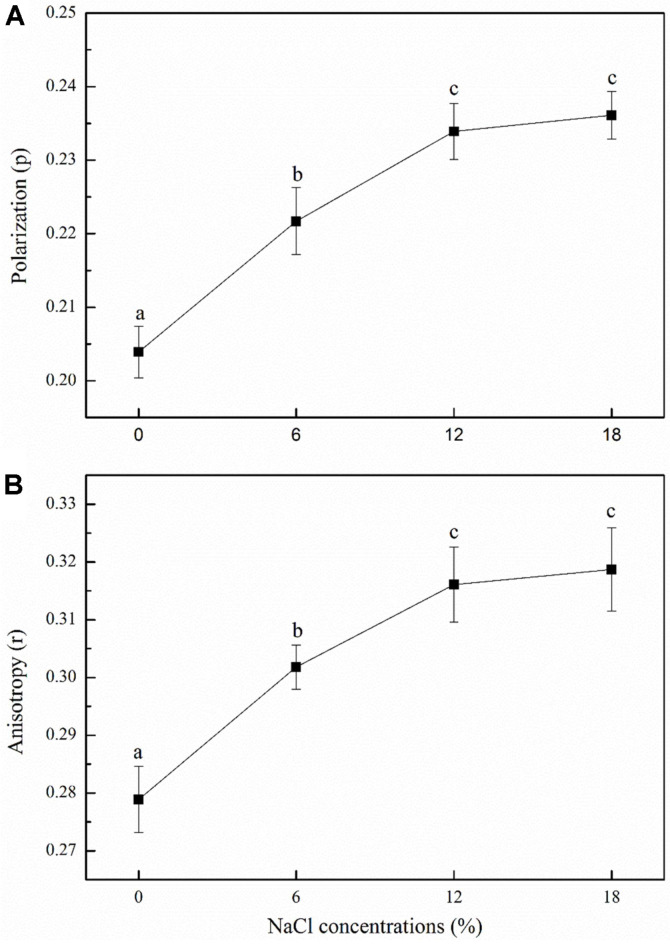
Changes in membrane fluidity of *Z. rouxii* under salt stress (NaCl concentration: 0%, 6%, 12%, and 18%). The degree of fluorescence polarization (**A**) and anisotropy (**B**) changes were measured by the spectrofluorometer. *Error bars*: SD (*n* = 3). Statistically significant differences (*p* < 0.05) were determined by one-way ANOVA with Duncan’s test and were indicated with different letters.

DPH is considered to be a sensitive fluorescent probe for studying the fluidity of membrane lipids because it can bind to their non-polar hydrocarbon chain. Fig. 5 presents the changes in cell membrane fluidity under salt stress. A significant increase in fluorescence anisotropy from 0.28 to 0.32 occurred when NaCl concentrations were increased from 0% to 18%. Meanwhile, the polarization also increased gradually from 0.20 to 0.24, and reached its maximum at 18% NaCl concentration. Generally, an increase in fluorescence anisotropy reflects a decrease in the fluidity of the lipid bilayer. Taken together, these results revealed that an increase in salt stress caused a decrease in cell membrane fluidity in *Z. rouxii*, and that this strategy protects cells from salt stress.

### Regulation of Membrane Fatty Acid Composition in *Z. rouxii* under Salt Stress

**Fig. 6:**
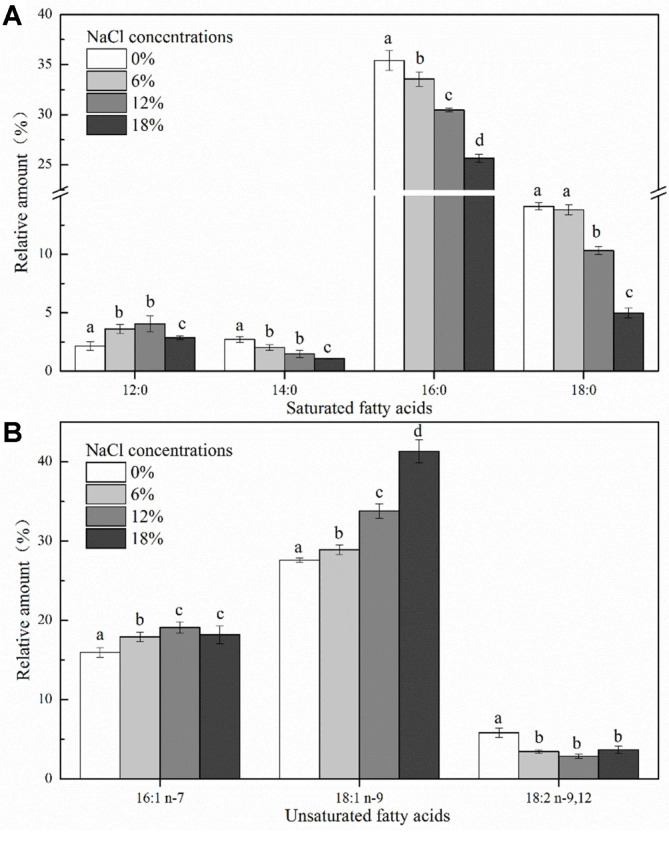
Alterations in membrane fatty acid proportions of *Z. rouxii* under salt stress (NaCl concentration: 0%, 6%, 12%, and 18%). Cells were cultured to mid-exponential growth phase under salt stress, and the proportion of membrane fatty acids including saturated fatty acids (**A**) and unsaturated fatty acids (**B**) were determined by GC-MS. The relative amount of FAMEs was calculated from peak areas. *Error bars*: SD (*n* = 3). Statistically significant differences (*p* < 0.05) were determined by one-way ANOVA with Duncan’s test and were indicated with different letters.

**Fig. 7:**
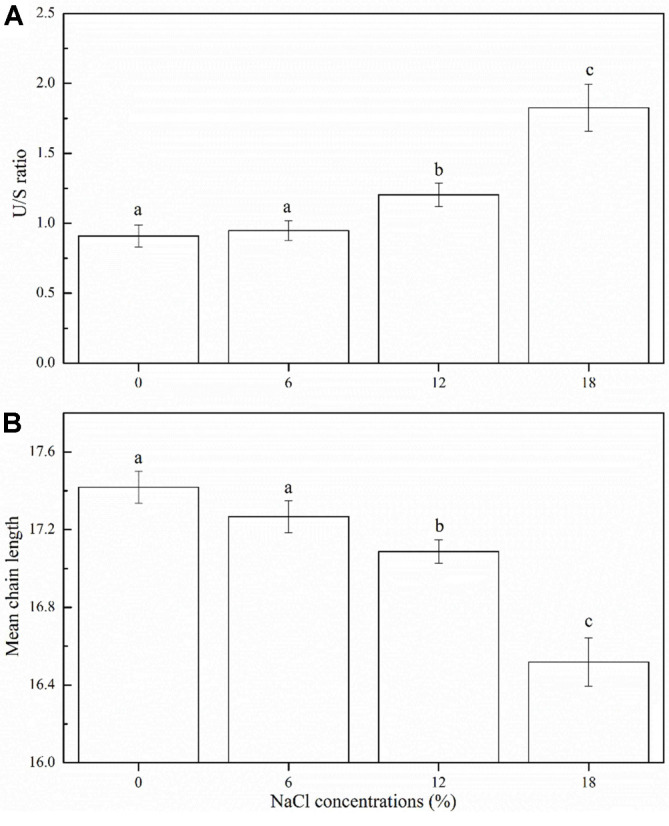
The unsaturation degree (**A**) and mean chain length (**B**) changes in membrane fatty acids of *Z. rouxii* under salt stress (NaCl concentration: 0%, 6%, 12% and 18%). The degree of unsaturation was calculated by unsaturated fatty acids/saturated fatty acids (U/S ratio). *Error bars*: SD (*n* = 3). Statistically significant differences (*p* < 0.05) were determined by oneway ANOVA with Duncan’s test and were indicated with different letters.

The fatty acids in cell membranes play an important role in maintaining membrane integrity and regulating the activity of membrane proteins. The effect of salt stress on cell membrane fatty acid composition is shown in Fig. 6: a total of 7 fatty acids, including 4 saturated fatty acids (lauric C12:0, myristic C14:0, palmitic C16:0, and stearic C18:0) and 3 unsaturated fatty acids (palmitoleic C16:1, oleic C18:1, linoleic C18:2), exhibited significant changes in their relative contents. In the absence of NaCl, the major cell membrane fatty acids of *Z. rouxii* were palmitic acid (C16:0), oleic acid (C18:1 n-9), and hexadecenoic acid (C16:1 n-7), which accounted for approximately 78% of the total fatty acid content. The contents of all saturated fatty acids (C14:0, C16:0, and C18:0, except C12:0) decreased with the increase in NaCl concentrations. In the case of unsaturated fatty acids, the proportions of hexadecenoic acid (C16:1 n-7) and oleic acid (C18:1 n-9) increased, whereas that of linoleic acid (C18:2) decreased. In addition, the U/S ratio and mean chain length were determined during salt stress (Fig. 7). With the NaCl concentration increasing to 18%, the U/S ratio increased by 101.21% and the mean chain length decreased by 5.16% compared with that observed in the absence of salts. It can be seen that the proportion of unsaturated fatty acids increased under salt stress, thereby resulting in a significant increase in U/S ratio.

## Discussion

In present study, we performed a comprehensive analysis of the cell membrane structure and functionally in *Z. rouxii* to elucidate its response to salt stress. TEM analysis revealed that under salt stress, the shape of *Z. rouxii* cells changed from round to elliptical and that some cell walls were ruptured, which was accompanied by intracellular substance spillover (Fig. 1). Similarly, Gandhi *et al*. [18] investigated the effects of salt stress on the morphology of *Lactobacillus acidophilus*, *Lactobacillus casei*, and *Bifidobacterium bifidum* by TEM, and found that at 10%NaCl concentration, the cell structures of all the bacteria were irregular with several deformities in the cell membranes. Therefore, the change in cell structure can be considered as a mean of adaptation of the cells to unfavorable environmental conditions.

Regarding intracellular Na^+^ and K^+^ concentrations (Fig. 2A), it was observed that Na^+^ concentrations increased with the increase in NaCl concentrations. The Na^+^/K^+^ ratio showed an increasing trend, and under salt stress, this ratio was significantly higher than that in the absence of salts (Fig. 2B). A similar result has been reported by Andreishcheva *et al*. [19], who showed that intracellular Na^+^ concentrations have a small but reproducible increase at 9% salt concentration. This may be explained by the fact that extracellular Na^+^ concentrations were much higher than intracellular Na^+^ concentrations, and Na^+^ was forced into the cell due to this concentration gradient. Conversely, the K^+^ concentrations decreased significantly from 36.9 ± 2.10 to 8.68 ± 2.86 μg/mg when NaCl concentrations increased from 0% to 18%. The significant decrease in K^+^ content may result from Na^+^ ions entering the cell, leading to an increase in intracellular pressure, which caused the cells to extrude K^+^ as a way to counterbalance [20]. Generally, regulation of the intracellular Na^+^/K^+^ ratio is a strategy to resist salt stress, and previous research has suggested that bacteria respond to hypertonic stress by Na^+^ exclusion and K^+^ uptake [21]. Maintaining a low Na^+^/K^+^ ratio would help cells survive under salt stress [22]. In the present study, the Na^+^/K^+^ ratio increased significantly at 18% NaCl concentration, thus suggesting that cells were severely damaged at this salt concentration. The Na^+^/K^+^-ATPase is a transmembrane protein that maintains the homeostasis of cells by regulating the intracellular Na^+^ and K^+^ gradient [23]. Most euryhaline organisms exhibit adaptive changes in Na^+^/K^+^-ATPase activity following salinity changes [24]. Moreover, Na^+^/K^+^- ATPase is a widely recognized biomarker for evaluating salinity adaptation. Petrezsélyová *et al*. [25] revealed that the up-regulation of the Na^+^/K^+^-ATPase Ena1 expression is a crucial event for adaptation to high salt stress in the budding yeast *S. cerevisiae*. In the p resent study, Na^+^/K^+^- ATPase activity increased and significant differences were observed between the salinity treatments (Fig. 3). Thus, it can be inferred that Na^+^/K^+^-ATPase activity was increased in *Z. rouxii* under salt stress to create electrochemical gradients that provide the driving force for ion transport. At 18% NaCl concentration, Na^+^-K^+^-ATPase activity decreased to 0.93 μmol/mg protein, which can be mutually corroborated with the considerably increasing Na^+^/K^+^ ratio.

Ergosterol is a sterol found in lower eukaryotic cell membranes, and it plays an important role in cell membrane fluidity and permeability. It has been reported that ergosterol maintains the structural integrity of yeast membranes under stressful environmental conditions [26, 27]. Abe *et al*. [28] analyzed the ability of ergosterol in adjusting the dynamic properties of plasma membranes, and showed that the *erg2* mutation decreased the membrane order parameter, and drastically increased the rotational diffusion coefficient of plasma membranes, thus providing evidence for the requirement of ergosterol for membrane integrity. In the present study, the ergosterol concentration gradually increased with the increase in the salt (NaCl) concentration (Fig. 4), which is consistent with the results reported in a previous study [29]. Swan *et al*. [30] revealed that cells with high levels of sterols were more tolerant to environmental stress, and that ergosterol concentrations were parallel to the survival data. Yu *et al*. [31] also found that ergosterol concentrations were 1.6-fold higher than parent strains, which enhanced the stress tolerance of *S. cerevisiae*. Therefore, it can be inferred that *Z. rouxii* increases the biosynthesis of ergosterol to maintain cell membrane stability and reduce the damage caused by salt stress. The biosynthesis of ergosterol is tightly regulated by 25 known enzymes of the ergosterol production pathway [32]. Zhang *et al*. [33] have revealed that the ergosterol synthesis genes in *Pichia pastoris*, such as *ERG2*, *ERG3* and *ERG6*, up-regulated under ethanol stress, indicating that unsaturated fatty acids and ergosterol act synergistically to some extent to protect the plasma membrane. Furthermore, Kamthan *et al*. [34] have found that an increase in ethanol concentrations and thermal tolerance in fission yeast can be attributed to a 1.5-fold increase in ergosterol and oleic acid concentrations, which are consistent with the results obtained in the present study.

Membrane fluidity, which is well known to be essential for cell function, is an important regulator of cellular responses to ambient environmental stress to maintain the biologically active state of cell membranes [35]. In the present study, changes in cell membrane fluidity were determined by the rotational diffusion of fatty acyl chains, which was determined by fluorescence anisotropy with DPH as a probe. DPH is considered to be a sensitive fluorescent probe for studying the fluidity of membrane lipids because it can be bind to the nonpolar hydrocarbon chain of membrane lipids. As expected, the findings of the present study showed that an increase in salt stress resulted in a decrease in cell membrane fluidity of *Z. rouxii*, which was consistent with the results reported in previous studies [36-38]. Murínová and Dercová [39] have revealed that in the presence of unfavorable compounds such as toxic agents, cell membrane fluidity decreases to prevent the entry of these toxic compounds into the cells. Therefore, it can be inferred that under salt stress, *Z. rouxii* cells decrease cell membrane fluidity to protect the cells by preventing the entry of excessive salts. In addition, the regulation of cell membrane fatty acid profiles is one of the effective methods for cells to combat environmental stress. Under high osmotic pressure, most cells maintain their biochemical function by altering the membrane components [40]. The results of cell membrane fatty acid analysis showed that the proportion of total UFAs increased, whereas that of SFAs decreased, thereby resulting in a 101.21% increase in the U/S ratio (Fig. 7). Similar results were reported in a study on *Debaryomyces hansenii* under salt stress [41], which showed that high NaCl concentrations result in an increase in the degree of unsaturation magnitude of fatty acids within the membrane. Based on these results, the interesting relationship between cell membrane fluidity and fatty acid composition must be discussed further. The extent of cell membrane fluidity is also affected by the changes in fatty acid composition within the membrane phospholipids. The changes in the fluidity of natural membranes are related to chemical kinetics [42]. The increase in the U/S ratio and mean chain length may contribute to maintaining cell membrane fluidity, because membranes comprising long chain fatty acids are densely arranged, whereas those comprised of UFA are loosely arranged and more fluid [43]. However, the results of the present study do not seem completely consistent with this mechanism, because the proportion of UFAs in *Z. rouxii* increased under salt stress, whereas the cell membrane fluidity and mean chain length decreased slightly (Figs. 5-7). This decrease in cell membrane fluidity may also be related to high salt concentrations. Qi *et al*. [36] have found that the decrease in cell membrane fluidity could restrict the influx of Na^+^ and increase Na^+^/K^+^-ATPase activity in response to salt stress.

In conclusion, we used a physiological approach in the present study to investigate the responses of *Z. rouxii* to salt stress at the membrane level using salt tolerance tests to reveal its protective mechanisms. The results of our study elucidate the tolerance mechanisms of *Z. rouxii* at high salt (NaCl) concentrations, which may be useful in the development of new strategies to increase the industrial utilization of this species under salt stress.
